# Mindfulness, Phenomenology, and Psychological Science

**DOI:** 10.1007/s12124-024-09841-z

**Published:** 2024-04-22

**Authors:** Lars-Gunnar Lundh

**Affiliations:** Department of Psychology, Lund University, Lund, Sweden

**Keywords:** Mindfulness, Phenomenology, Experimental phenomenology, Creativity, Theoretical phenomenology, Psychological science

## Abstract

Most present-day research on mindfulness treats mindfulness as a variable that is studied in relation to other variables. Although this research may provide us with important knowledge at the population level and mechanism level, it contributes little to our understanding of the phenomenon of mindfulness as it is experienced and enacted at the person level. The present paper takes a person-oriented phenomenological perspective on mindfulness, comparing this perspective with that of von Fircks’ (2023). In a first part of the paper, mindfulness is discussed as a phenomenological practice that can be studied by means of experimental phenomenology. It is argued that there is room for the development of an immense variety of personalized mindfulness practices that may serve people’s health and well-being. The second part of the paper contains a brief discussion of the possible role of mindful observation and reflection in psychological research. It is argued that mindfulness skills may be important both for improving the quality of phenomenological observation and to facilitate creative thinking in connection with the development of psychological theory. A main implication is that an integration between mindfulness and phenomenology may serve as an important part of this process.

## Introduction

*Mindfulness deserves to be treated as more than just a variable in psychological research.* (von Fircks, 2023)

Most of present-day research on mindfulness treats mindfulness as a variable that is studied in relation to other variables. All too seldom is there a focus on the *phenomenon* of mindfulness and different variations of mindfulness, and what it means for a person to be mindful. This is not to deny the value of variable-oriented research. Such research can provide us, for example, with important information about treatment effects at the population level (i.e., the percentage of individuals who benefit from a mindfulness-based treatment). It may also provide us with psychometric instruments to measure degree of mindfulness, and knowledge about the neural mechanisms underlying mindfulness. What it fails to provide us with, however, is an increased understanding of the role of mindfulness at a person-level.

In a previous paper (Lundh, 2023) I have argued that psychological science has three main branches, corresponding to three levels of research: (1) research at the person level; (2) research at the population level, and (3) research at the mechanism level:

- *Person*-level research focuses on psychological phenomena as experienced and enacted by individual persons, in their interaction with other persons and the environment, and as developing over time.
- *Population*-level research focuses on *populations of* individuals, frequencies of various psychological phenomena in a population, risk factors, and population-level effects of various psychological interventions.
- *Mechanism*-level research focuses on psychological functioning as explained in terms of mechanisms at *a sub-personal* level (neurophysiological mechanisms, information processes).

Although the person-level might be expected to form a core of psychological science, it has a rather marginal role in present-day research. Sad to say, this applies also to research on mindfulness. It is therefore refreshing to read von Fircks’ (2023) paper. In the present paper I will discuss some of the themes in his paper from the perspective of a person-oriented phenomenological paradigm. Although my perspective differs from that of von Fircks, the two perspectives converge in several ways.

First, some words about phenomenology. The term phenomenology has been used in many ways, with different meanings, but here it is defined as *the scientific analysis of our experience of being in the world*, and a differentiation is made between experimental phenomenology and theoretical phenomenology (Lundh, 2020). The present paper contains two main sections. The first section contains a discussion of mindfulness from an experimental-phenomenological perspective. The second section focuses on the possible role of mindful observation and reflection in theoretical phenomenology.

## Experimental Phenomenology: The Study of Phenomenological Practices

*There is a multitude of different types of mindfulness* (von Fircks, 2023)

Experimental phenomenology is the study of phenomenological practices (Lundh, 2020, 2022a). Phenomenological practices represent a large category of *intentional variations of experiencing*, defined in terms of choice of *attitude* and direction of *attention*. Because there is a wide variety of attitudes that can be taken to our experiences, and likewise a wide variety of possible objects of attention, this leaves room for a multitude of different phenomenological practices. Some of these, but not all of them, deserve the name of mindfulness practices.

There are many different attempts to define mindfulness in the literature, but they all mention at least two components: (1) *attending* to present experience, (2) with a particular kind of *attitude*. The kind of attitude is described variously as being accepting and non-judging; kind, friendly and caring; and showing openness, curiosity, and non-reactivity to experience (e.g., Kabat-Zinn, 2013; Shapiro & Carlson, 2017). Although mindfulness means to attend to *present* experience, there is a large variety of aspects of present experience that can be attended.

Experimental phenomenology is experimental in a double sense. First, it expresses an *experimental attitude* by developing and testing new variations of phenomenological practices. Second, it intends to study the *effects* of phenomenological practices by experimental methods (e.g., single-subjects designs). It differs from traditional experimental research on *standardized* mindfulness programs that are administered in a group format, such as Mindful-Based Stress Reduction (MBSR; Kabat-Zinn, 2013), by involving *personalized* practices that are tailor-made for the individual and guided by an individualized set of self-instructions. The focus of attention and the choice of attitude are decided in collaboration between patient and therapist, to suit the needs of the individual. The participant is also instructed to keep track of experiences during and after practice, either in the form of quantitative ratings or in the form of diary notes.

What makes experimental phenomenology into a scientific endeavor is the intersubjective nature of this kind of study. First, each type of phenomenological practice is characterized by specific attitudes and a specific direction of attention; and because these are formulated as verbal self-instructions they can be *taught* and *trained* and transferred from one person to another. Second, potential *effects* described by one person can be subjected to *replication* both by the same person, and by other persons. This also means that the conclusions from such a study are hypothetical and provisional and can be modified or specified as the result of further investigations.

The following three examples illustrate the wide variety of mindfulness practices that are possible within this paradigm. First a mindful embodiment practice is described that was developed for a patient suffering from insomnia. The second example describes a mindful driving practice used by a participant who suffered from daytime sleepiness. The third example is a mindful gratitude practice developed for a person who desired a spiritually oriented exercise. A comparison between these three practices also illustrates how experimental phenomenology has ambitions to generalize theoretically beyond specific practices.

### The Mindful Embodiment Practice (MEP)

This practice is a short variant of the body scan as used in standardized mindfulness programs (e.g., Kabat-Zinn, 2013; Shapiro & Carlson, 2017). The full form of the body scan typically involves (1) having one’s attention slowly moving systematically through every region of the body; and (2) “breathing into” each body area in connection with attending to it. The short MEP as described below included only four body parts: the eyes (and the area around the eyes), the lips, the neck, and the chest.

The instructions were developed in collaboration with the patient. When unable to fall asleep, the patient was to stay in bed and practice the MEP, according to the following self-instructions, which he liked to describe as a “poem” with four verses, where the first verse focused on the eyes:

*May I explore*

*the feelings in my eyes*

*by breathing into*

*that part of my body*

*slowly*

The following three “verses” repeated the same instruction with regards to the lips, the neck, and the chest, in that order. The case is described in detail by Lundh (2022b).

### Mindful Driving

This practice was developed in close collaboration with a patient who had problems with sleepiness during driving, and the self-instructions were tested successively during the driving of shorter distances before arriving at their final formulations. The rationale for this practice involved statements such as “driving, like everything else, affords a valuable and exciting opportunity for mindful experiencing”. The practice involved four phases with different attentional foci: (1) the road; (2) the traffic flow; (3) the landscape; and (4) the sitting (for a detailed description of the self-instructions, see Lundh [2020, p. 500–501]). The patient successively learned these instructions by heart, to be able to shift freely between the four different phases according to the traffic situation and other circumstances.

### The Mindful Gratitude Practice

This practice (described by Lundh, 2024) was developed together with a client who desired a spiritually oriented nature-focused exercise free from traditional religious doctrines. The original version of the practice had the following self-instructions:

*Explore* how it feels to breathe. Notice the air going in and out. Note how the breathing *connects* me with the surrounding world, makes me inseparable from my surroundings. *Thanks* for the air that surrounds me, the air that I breathe, that gives me life. Thanks for the lungs that make me able to breathe.

*Explore* the sights in front of me, with all the different shades of colour and shapes of things. Note how the light *connects* me with the surrounding world, makes me inseparable from my surroundings. *Thanks* for the light that presents me with all these things, and thanks for my eyes that make me able to see.

*Explore* the sounds around me, the different sounds that come and go. Note how the sounds *connect* me with the surrounding world, make me inseparable from my surroundings. *Thanks* for the air that passes all these sounds to me, and thanks for my ears that make me able to hear.

*Explore* the feelings in my skin and in my whole body. Note all the sensations that *connect* me with my body, make me inseparable from my body. Thanks for the body that gives me access to the world in my own particular way.

### From Idiographic Description to Nomological Generalization

Although experimental phenomenology is primarily an idiographic kind of research, it does not stay at the idiographic level but also harbours a nomological ambition to arrive at general theoretical principles. This may be illustrated by comparing the three phenomenological practices described above.

First, it may be noted that the two first practices were intended to have very different effects, the MEP facilitating sleep, and the mindful driving practice facilitating alertness. This gives a nice illustration of the wide variety of mindfulness practices, not only in terms of their contents but also with regards to their intended *effects*. Someone may wonder how mindfulness training can facilitate sleep in one case and alertness in another case – that is, two apparently opposite effects. The suggested answer is that what matters is the direction of attention: In MEP the attention is turned *inwards to the body*, whereas in mindful driving the attention is turned *outwards* to the surroundings and one’s active behavior in relation to these surroundings. Attention turned inwards to the body, at least under certain conditions, leads to *cognitive deactivation* (thereby facilitating sleep), whereas attention turned outwards to one’s active transactions with the surrounding is more likely to lead to *cognitive activation* (thereby promoting alertness).

Being mindful is being attentive to aspects of present experience, but there is an enormous range of possible aspects that can be attended to, and the effects are likely to differ depending on the direction of attention. In the third example above, the mindful gratitude practice, attention is focused on (1) an exploration of sensory experiences; (2) experiences of connectedness with the surrounding world; and (3) feelings of gratitude. Experiences that have been reported as the result of this practice are experiential insights about the “ecological” nature of one’s being in the world, and feelings of wonder and thankfulness.

Here it does not seem too far-fetched to conclude that experimental-phenomenological research may contribute to the development of “an organic conceptual system that could help people to better practice mindfulness in their daily lives”, to borrow a formulation from von Fircks (2023).

### Meta-mindfulness

*the principle of* wu wei*…is often mis-translated as the art of non-doing… a more appropriate translation would be the art of not-forcing.* (von Fircks, 2023)

As argued above, mindful attention is always *selective.* This raises the question how the selection is done. This may be referred to as *meta-mindfulness regulation*. Langer (2014) speaks of it in terms of “second-order mindfulness”, defined as “choosing what to be mindful about” (p. 197). Lutz et al.’s (2008) distinction between focused attention meditation (FAM) and open monitoring meditation (OMM) can be seen as a distinction between two varieties of meta-mindfulness regulation. FAM requires practitioners to focus their attention on a specific object or event, whereas the practitioner in OMM is to remain attentive to any experience that might arise, without intentionally selecting any particular object.

Even FAM, however, involves an attitude of “non-forcing”. This is illustrated by the instructions that are typically given to mindfulness practitioners when they get distracted by various kinds of thoughts. The typical instruction is just to notice that one was distracted and then to *gently bring attention back* to the assigned focus (e.g., Kabat-Zinn, 2013). That is, although there is a directive attitude involved here, it is *not* characterized by forcing oneself back on track.

Another common characteristic of the three practices that were described above is that they were developed in close collaboration with the practitioner. This means that these practices are more likely to be experienced as *intrinsically meaningful*, which may facilitate a motivation to engage in the practice on one’s own. This illustrates another aspect of a non-forcing attitude.

## Theoretical Phenomenology as Mindful Reflection on Experience

The previous section was about the experimental-phenomenological study of mindfulness practices. In this final section, I will widen the perspective to the possible role of mindfulness in the theoretical-phenomenological study of psychological phenomena. Whereas experimental phenomenology is an investigation of the effects of phenomenological practices, theoretical phenomenology makes use of phenomenological observation and reflection to develop our theoretical understanding of our experience of being in the world. Here I will argue that mindfulness may be of help both for phenomenological observation and phenomenological reflection.

### The Role of Mindfulness in Phenomenological Observation

According to Husserl (1938/1970), to use a phenomenological method means to *shift perspective* from our usual “natural attitude” with its focus on the world, and our practical engagement with things in the world, to a phenomenological attitude characterized by a focus on our *experience of being in the world*. He refers to this as “epoché”, but partly similar concepts are found in the mindfulness literature, such as “decentering”, “meta-cognitive awareness”, and “cognitive defusion”. Common to these concepts is that they refer to the “capacity to shift experiential perspective—from *within* one’s subjective experience *onto* that experience” (Bernstein et al., 2015, s. 599). Another aspect of this change in perspective is that it involves a shift of focus from *doing* to *being*.

The ability to take a phenomenological attitude to experience lies at the basis of our capacity for phenomenological observation. Importantly, this suggests the possibility that the *training* of a phenomenological attitude may make people into more *skilled* phenomenological observers. If mindfulness meditation leads to an increased ability to attend to experience in an explorative and accepting way (i.e., in taking a phenomenological attitude), it may serve as a training in phenomenological observation skills. Evidence for this has been reported by Fox et al. (2012), who found that expert meditators showed significantly better introspective accuracy than novices, and that overall meditation experience significantly predicted individual introspective accuracy.

Importantly, phenomenological observation cannot be reduced merely to introspection, as it also includes a category of *general phenomenological observations*. General phenomenological observations are observations about our experience of being in the world that are in principle available to anyone of us (Lundh & Foster, 2024), and are therefore open to intersubjective evaluation. This can be illustrated by Husserl’s (1912/1989) analyses of the body. One simple example is Husserl’s observation that the perception of our own body as an object differs from our perception of other objects because it is typically “restricted in a definite way: certain of my corporal parts can be seen by me only in a peculiar perspectival foreshortening, and others (e.g., the head) are altogether invisible to me” (Husserl (1912/1989), p. 167). In fact, we have less direct perceptual access to our own body as an object than to other objects in the surrounding world (which we can walk around and inspect from all kinds of angles).

The reason for this, of course, is that we simply cannot step out of our own body to observe it from an external point of view. We perceive the world (including our own body) from the perspective given by the position of our body. As a theoretical extension, this kind of phenomenological reflection invites the concept of *embodiment* (Lundh & Foster, 2024): we do not only *have* a body, we also *are* this body, and *being* this body prevents us from fully observing the body we have.

This at the same time illustrates how phenomenological observation is tied to phenomenological *reflection*. Phenomenological reflection involves a synthesis of different observations. For example, to understand the nature of our bodily experience we need to synthesize a variety of phenomenological observations of how we not only perceive our own body as an object in the world, but also experience ourselves as *being* this body and as sensing it “from within” (Lundh & Foster, 2024). At an even higher level of abstraction, a variety of phenomenological observations may be synthesized theoretically into more integrative forms of theory. Further, as argued below, there is reason to believe that mindfulness may be of help also in this higher-order theoretical reflection.

### The Role of Mindfulness in Theoretical Reflection

Edmund Husserl was a pioneer in theoretical phenomenology. He referred to this as “transcendental phenomenology” and argued that it could supply us with the conceptual foundations of a psychological science, its “a priori structural concepts” (Husserl, 1938/1970, p. 260). A recurrent theme in his writings is that empirical psychology needs phenomenology in a similar way to that in which physics needs mathematics. A task of transcendental phenomenology, he argued, is to supply us with an understanding of the essential structures of psychological phenomena – an understanding that may serve as the basis for meaningful empirical research on psychological issues. This is an undertaking that involves not only theoretical analyses but also theoretical *syntheses* – that is, the conceptual integration of various observations and insights.

It is possible that a mindful attitude may be of help in the creative part of this kind of work. Experimental findings (Colzato et al., 2012) indicate that mindfulness meditation, especially open monitoring meditation (Lutz et al., 2008), is conducive to the kind of divergent thinking that is associated with creativity (Guilford, 1950). Other relevant concepts here are cognitive flexibility and mind wandering. According to Preiss (2022), the ability to alternate between controlled and spontaneous cognition is central to the kind of cognitive flexibility that characterizes creative thinking; in other words, creativity is associated with an ability to flexibly focus or defocus one’s attention depending on the nature of the task. Mind wandering represents spontaneous cognition and involves a “defocusing” of one’s attention. By reducing cognitive control, as Preiss argues, mind wandering can facilitate the emergence of new associations and generate unexpected solutions. Although mind wandering might seem to be the very opposite of mindful attention, there may in fact be such a thing as *mindful mind wandering* (Preiss, 2022, p. 2). This would occur when individuals, as part of their meta-mindfulness regulation, shift from concentrating on a certain topic to mindfully letting thoughts come and go, in an openness to novel juxtapositions of thoughts that deserve to be focused on as a way to reach new and potentially fruitful syntheses of previously unrelated ideas.

## Conclusion

The concept of mindfulness, taken in its widest sense and not tied to any specific religious context (e.g., Langer, 2014; Lundh, 2022c), may turn out to be an important integrative concept in psychological science. Not only is there room for the development of an immense variety of mindfulness practices that may serve people’s health and well-being. It is also possible that mindfulness skills may have role to play in the development of psychological theory, by means of improvements in phenomenological observation and reflection. A main implication of the reasoning in the present paper is that an integration between mindfulness and phenomenology may serve as an important part of this process.

## Data Availability

No datasets were generated or analysed during the current study.
